# Supplementary material for: High rates of evolution preceded shifts to sex-biased gene expression in Leucadendron, the most sexually dimorphic angiosperms
Source: eLife. 2021 Nov 2;10:e67485. doi: 10.7554/eLife.67485 (PMC8635981; doi:10.7554/eLife.67485)
Supplement: Supplementary file 2. [file elife-67485-supp2.pdf]

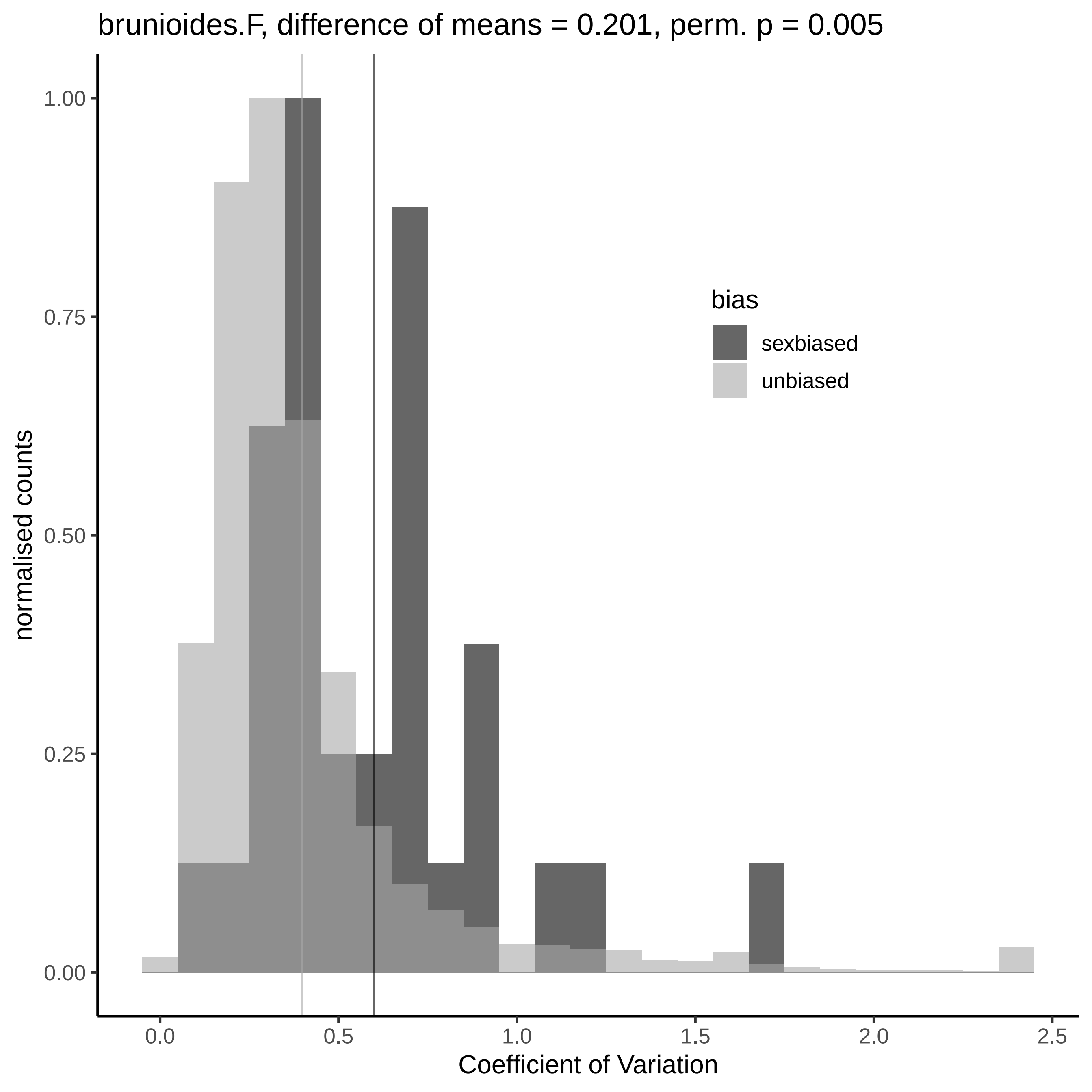
brunioides.F, difference of means = 0.201, perm. p = 0.005
normalised counts
1.00
0.75
0.50
0.25
0.00
0.0
0.5
1.0
1.5
2.0
2.5
Coefficient of Variation
bias
sexbiased
unbiased

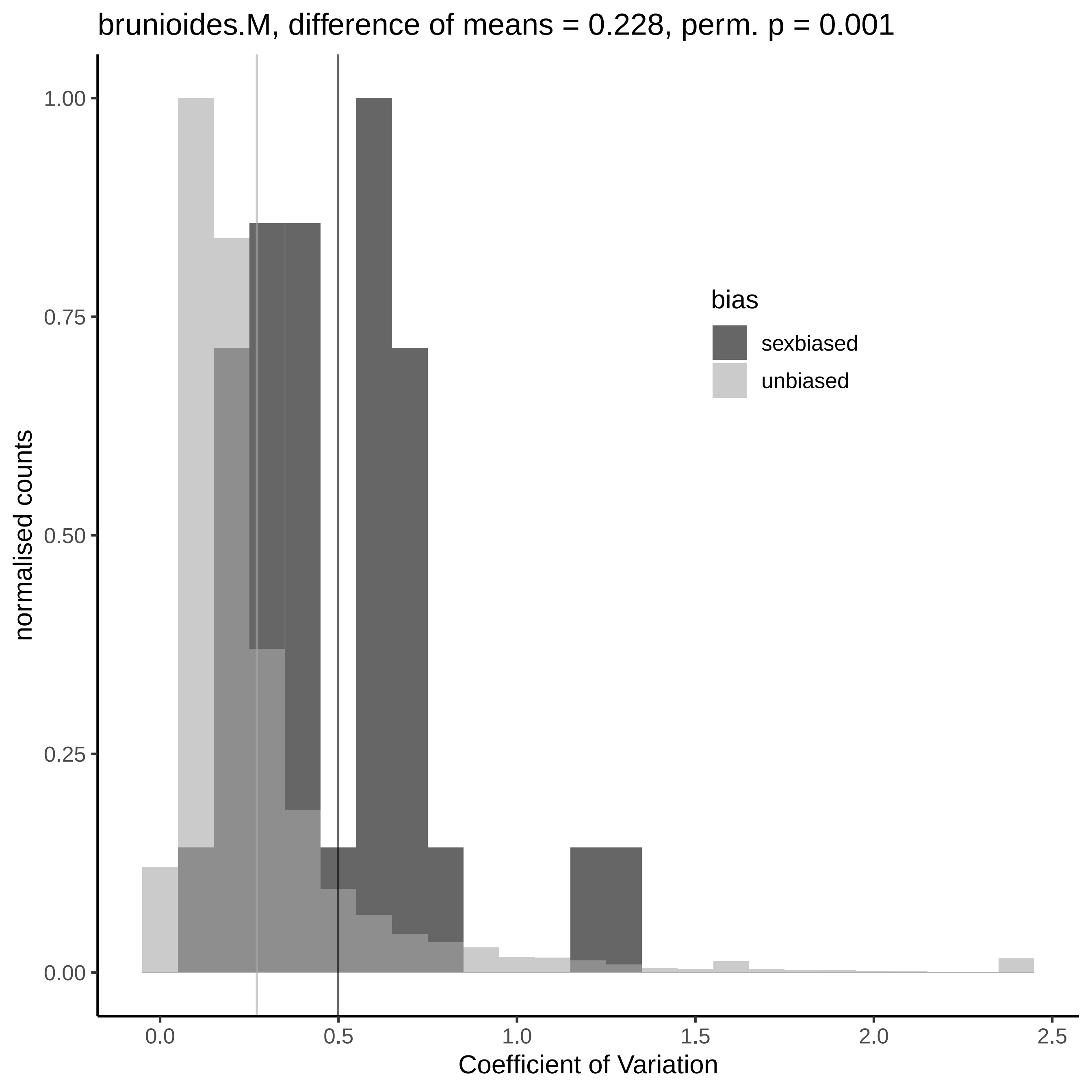
brunioides.M, difference of means = 0.228, perm. p = 0.001
normalised counts
1.00
0.75
0.50
0.25
0.00
0.0
0.5
1.0
1.5
2.0
2.5
Coefficient of Variation
bias
sexbiased
unbiased

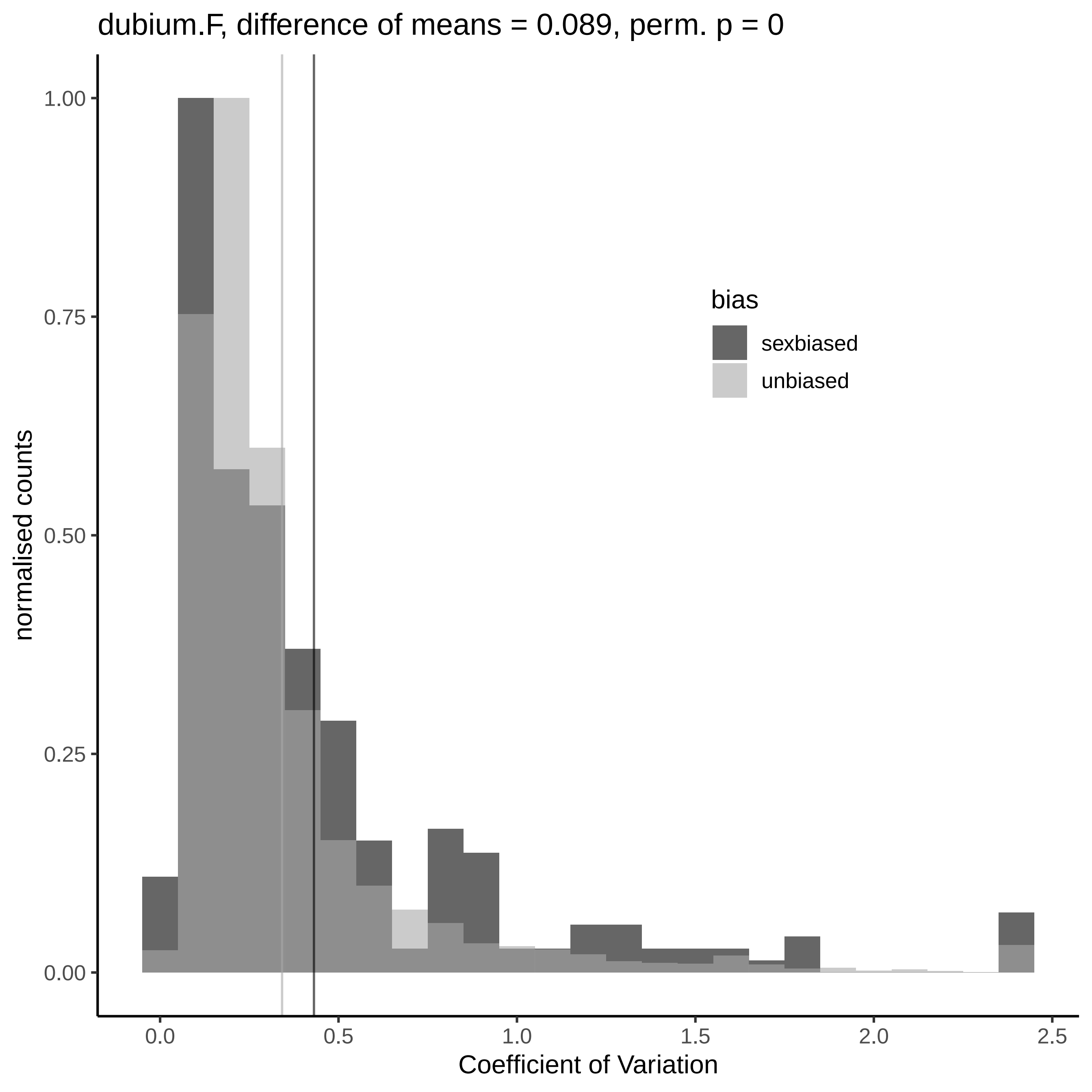

dubium.F, difference of means = 0.089, perm. p = 0
normalised counts
1.00
0.75
0.50
0.25
0.00
0.0
0.5
1.0
1.5
2.0
2.5
Coefficient of Variation
bias
sexbiased
unbiased

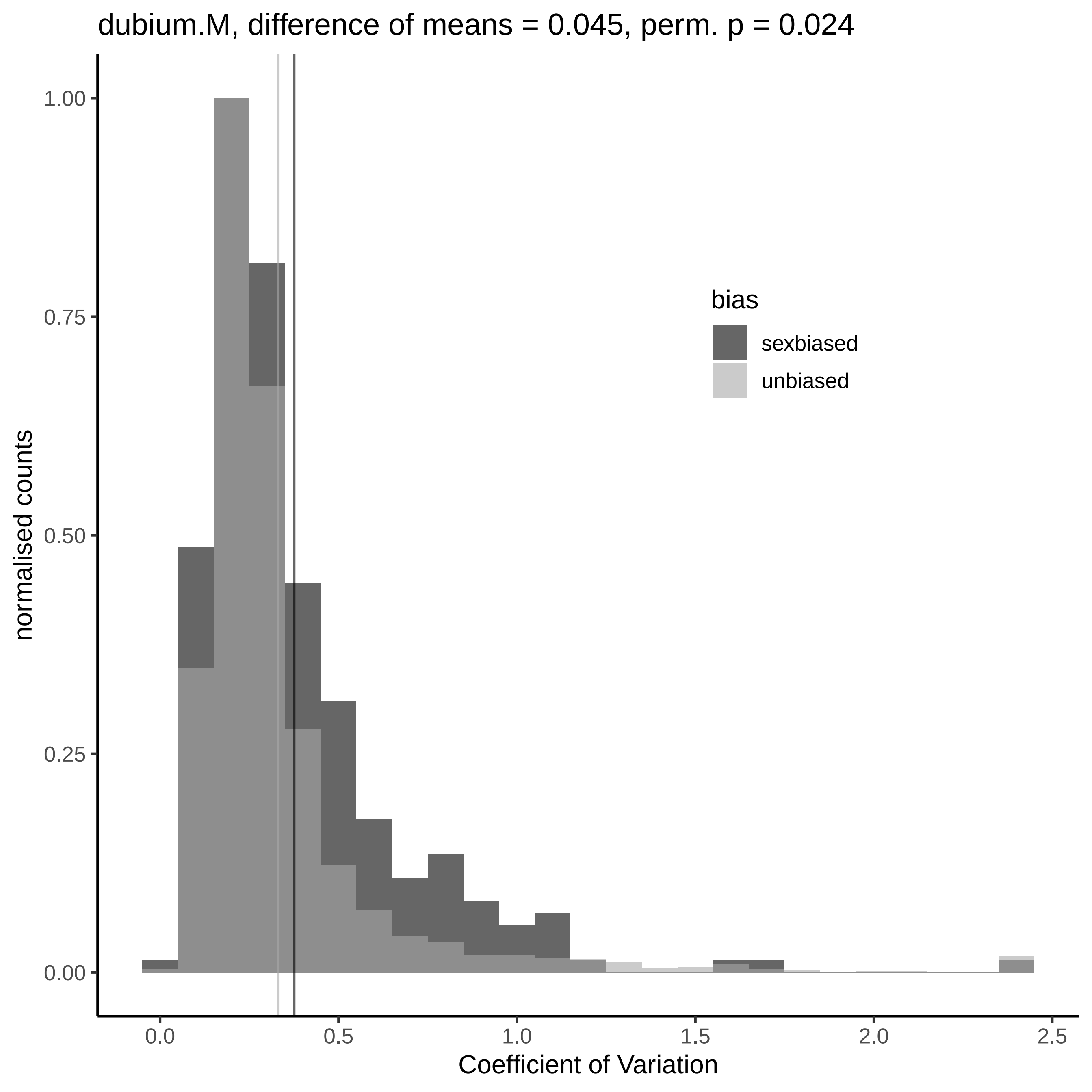
dubium.M, difference of means = 0.045, perm. p = 0.024
normalised counts
1.00
0.75
0.50
0.25
0.00
0.0
0.5
1.0
1.5
2.0
2.5
Coefficient of Variation
bias
sexbiased
unbiased

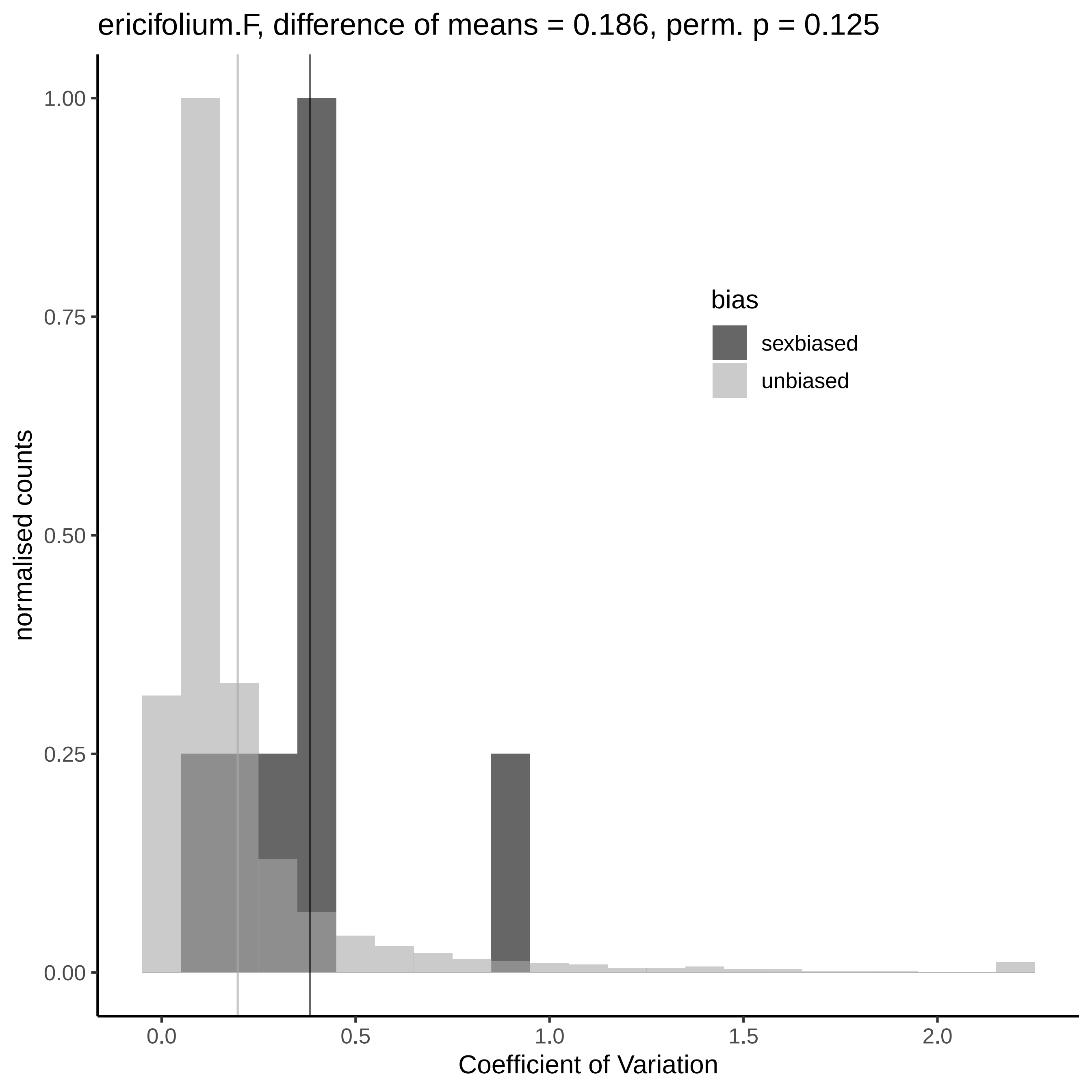
ericifolium.F, difference of means = 0.186, perm. p = 0.125
bias
sexbiased
unbiased
normalised counts
1.00
0.75
0.50
0.25
0.00
0.0
0.5
1.0
1.5
2.0
Coefficient of Variation

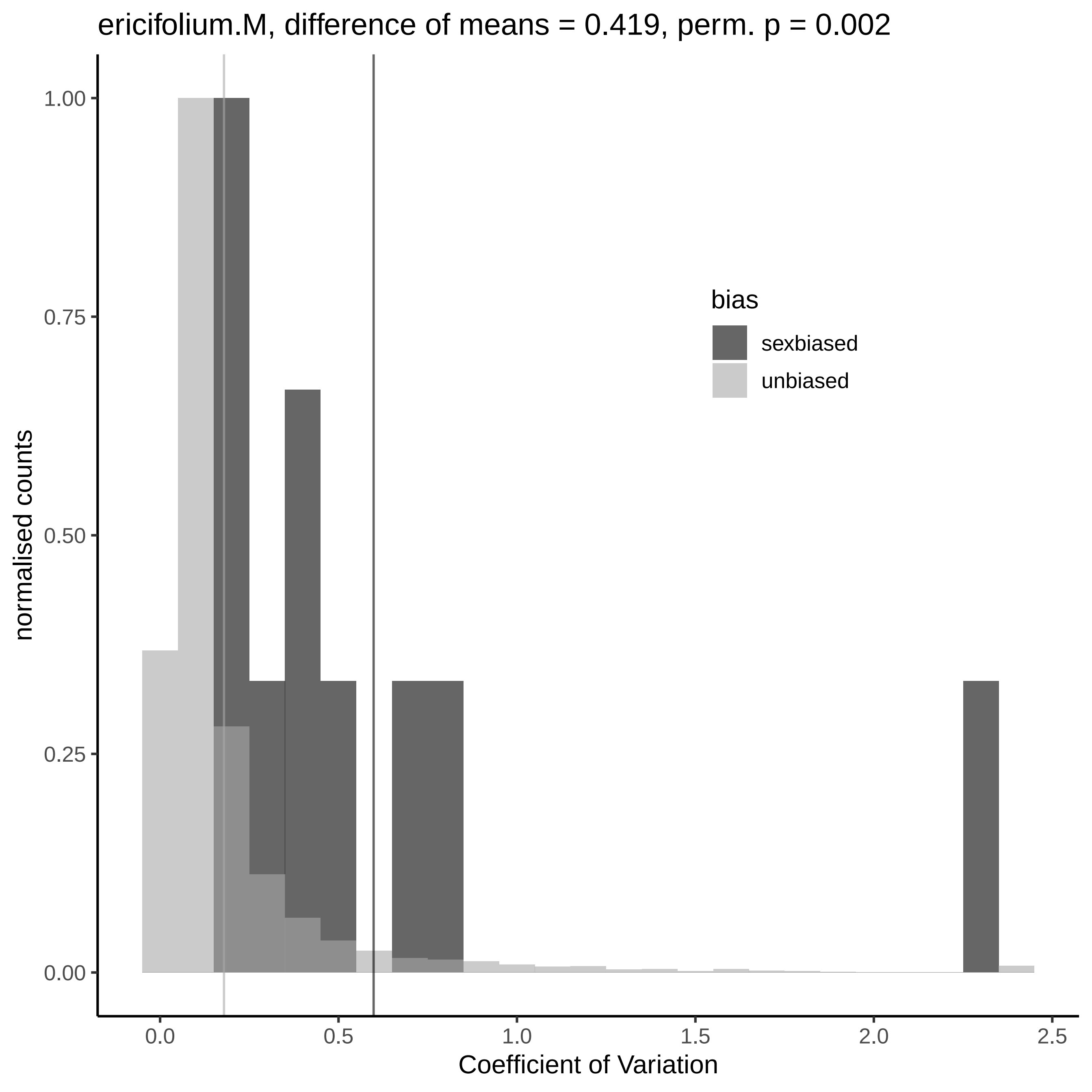
ericifolium.M, difference of means = 0.419, perm. p = 0.002
bias
sexbiased
unbiased
normalised counts
1.00
0.75
0.50
0.25
0.00
0.0
0.5
1.0
1.5
2.0
2.5
Coefficient of Variation

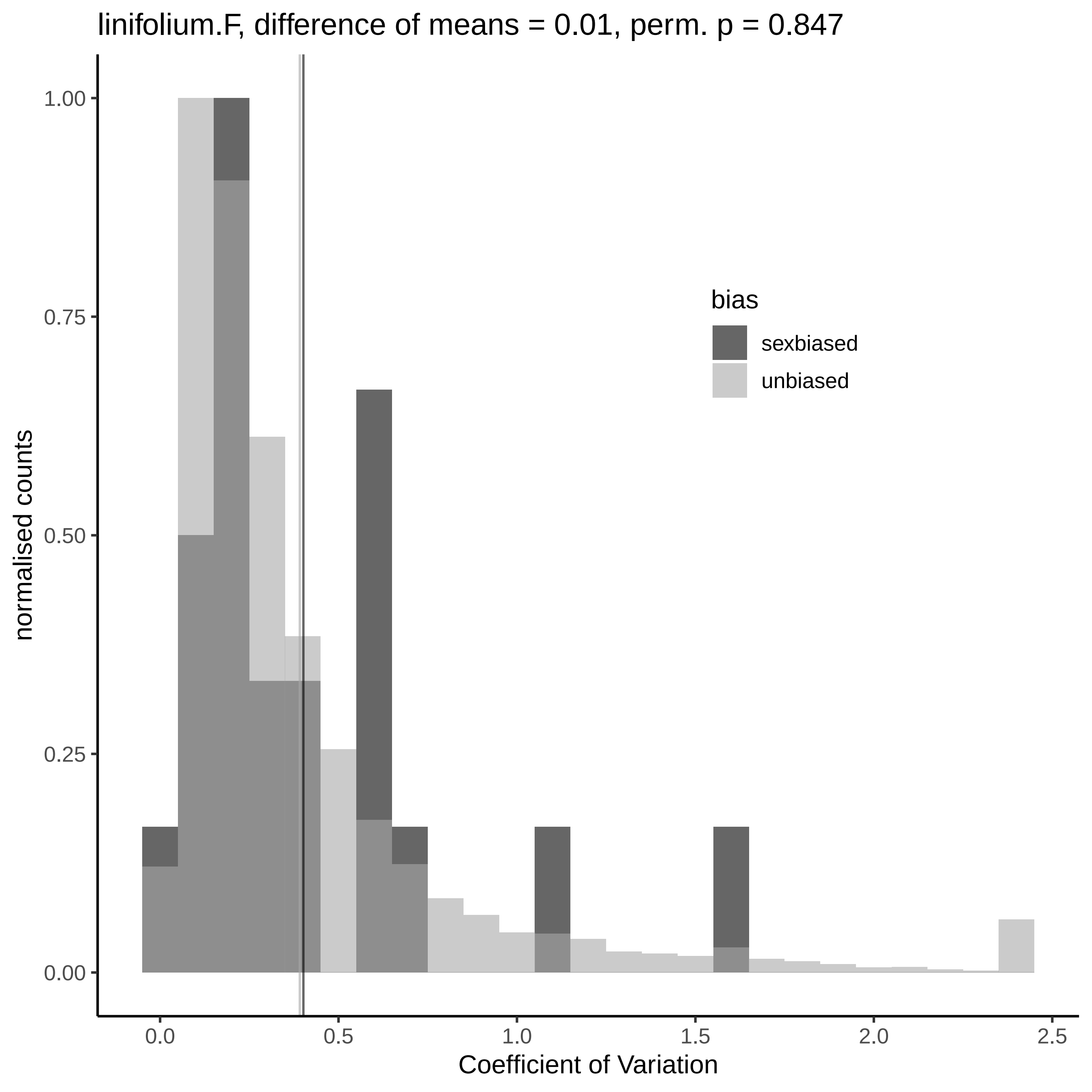
linifolium.F, difference of means = 0.01, perm. p = 0.847
normalised counts
1.00
0.75
0.50
0.25
0.00
0.0
0.5
1.0
1.5
2.0
2.5
Coefficient of Variation
bias
sexbiased
unbiased

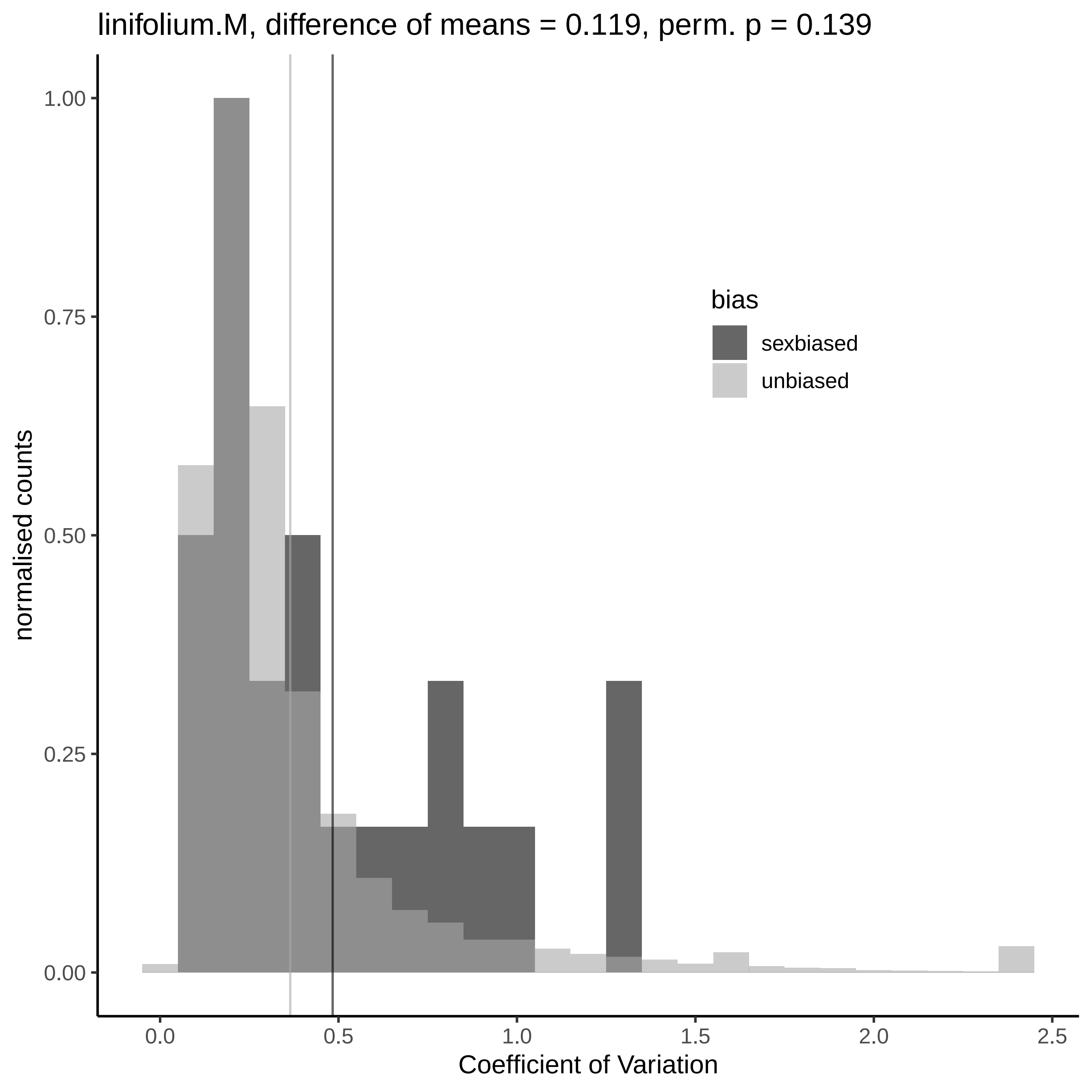
linifolium.M, difference of means = 0.119, perm. p = 0.139
bias
sexbiased
unbiased
normalised counts
1.00
0.75
0.50
0.25
0.00
0.0
0.5
1.0
1.5
2.0
2.5
Coefficient of Variation

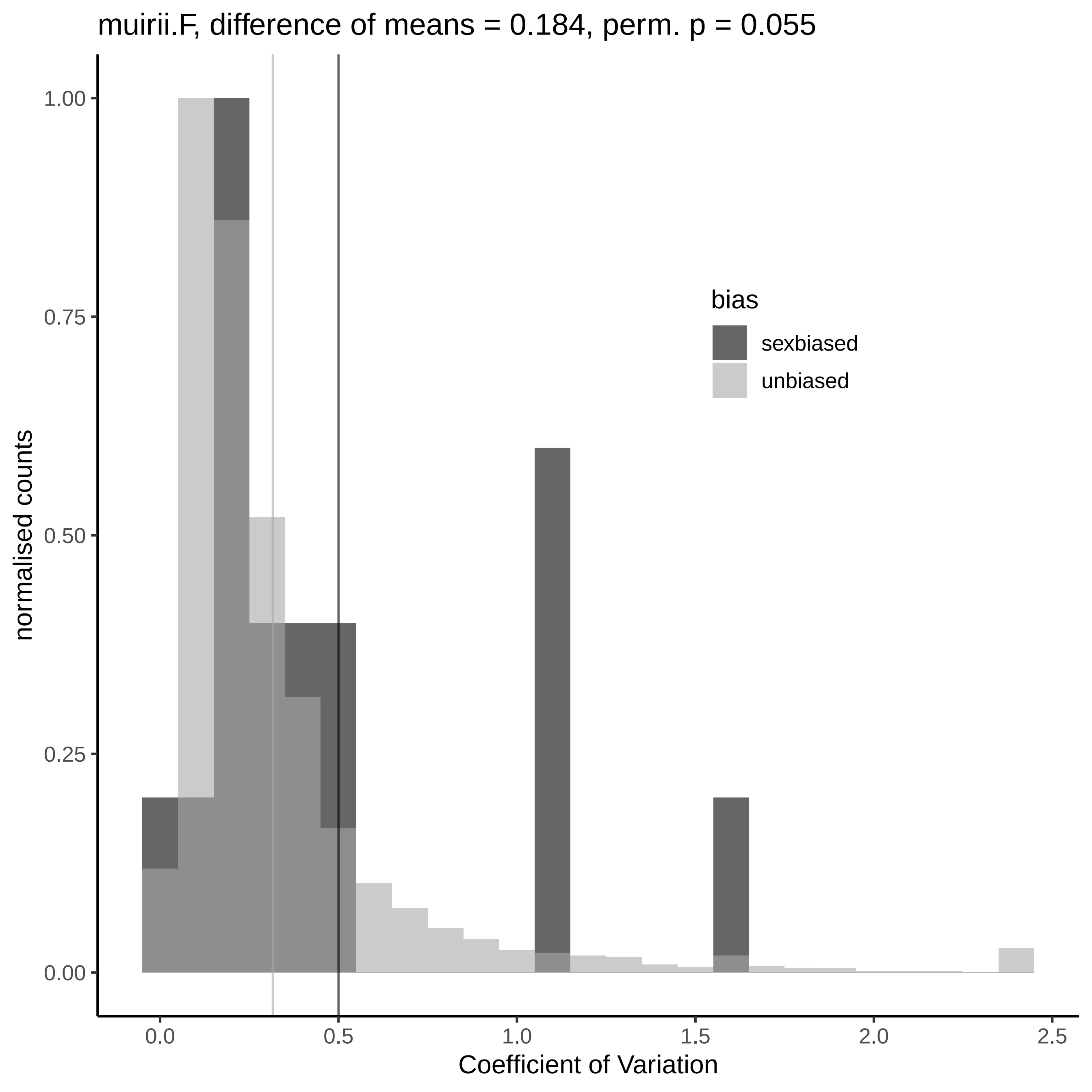
muirii.F, difference of means = 0.184, perm. p = 0.055
normalised counts
1.00
0.75
0.50
0.25
0.00
0.0
0.5
1.0
1.5
2.0
2.5
Coefficient of Variation
bias
sexbiased
unbiased

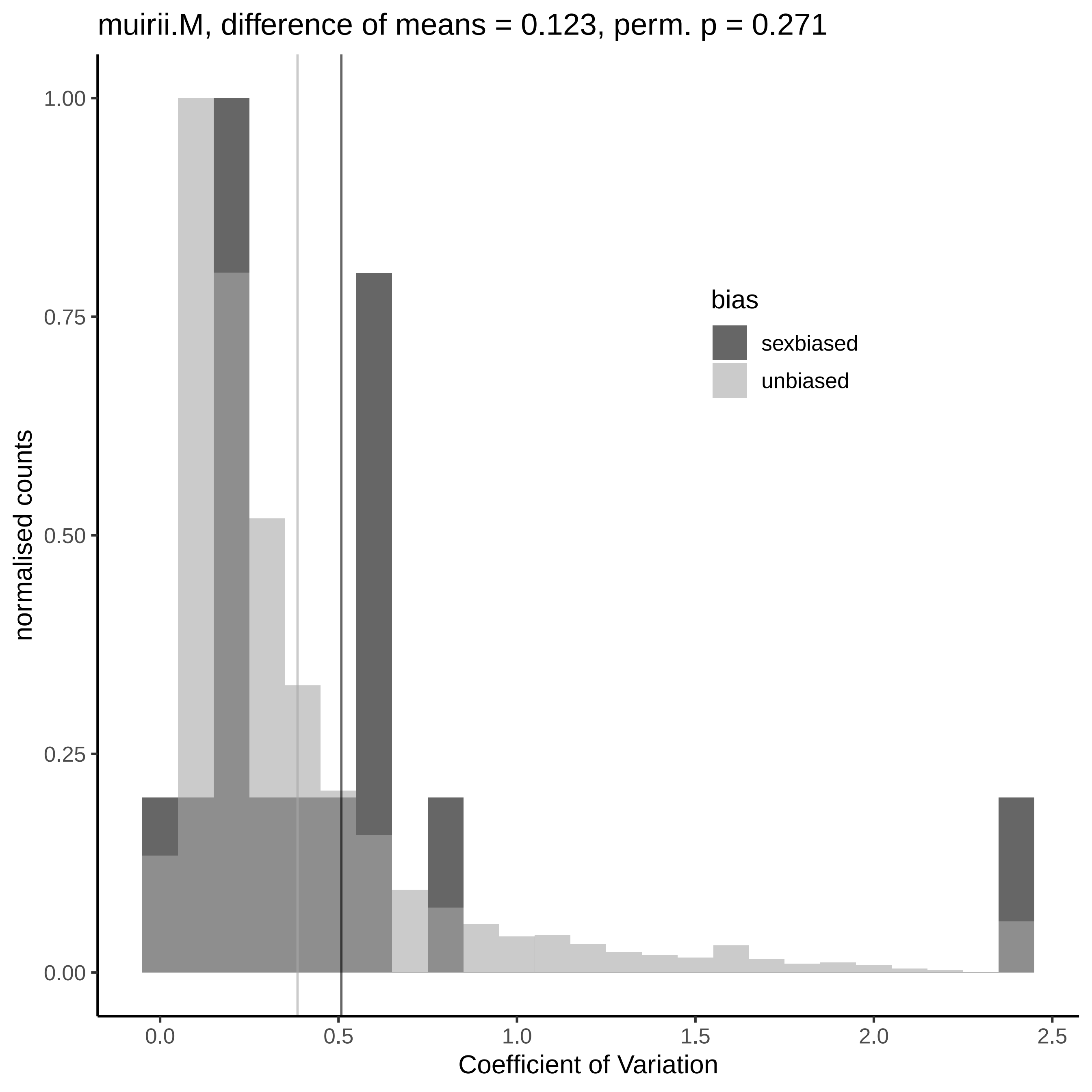

muirii.M, difference of means = 0.123, perm. p = 0.271
normalised counts
1.00
0.75
0.50
0.25
0.00
0.0
0.5
1.0
1.5
2.0
2.5
Coefficient of Variation
bias
sexbiased
unbiased

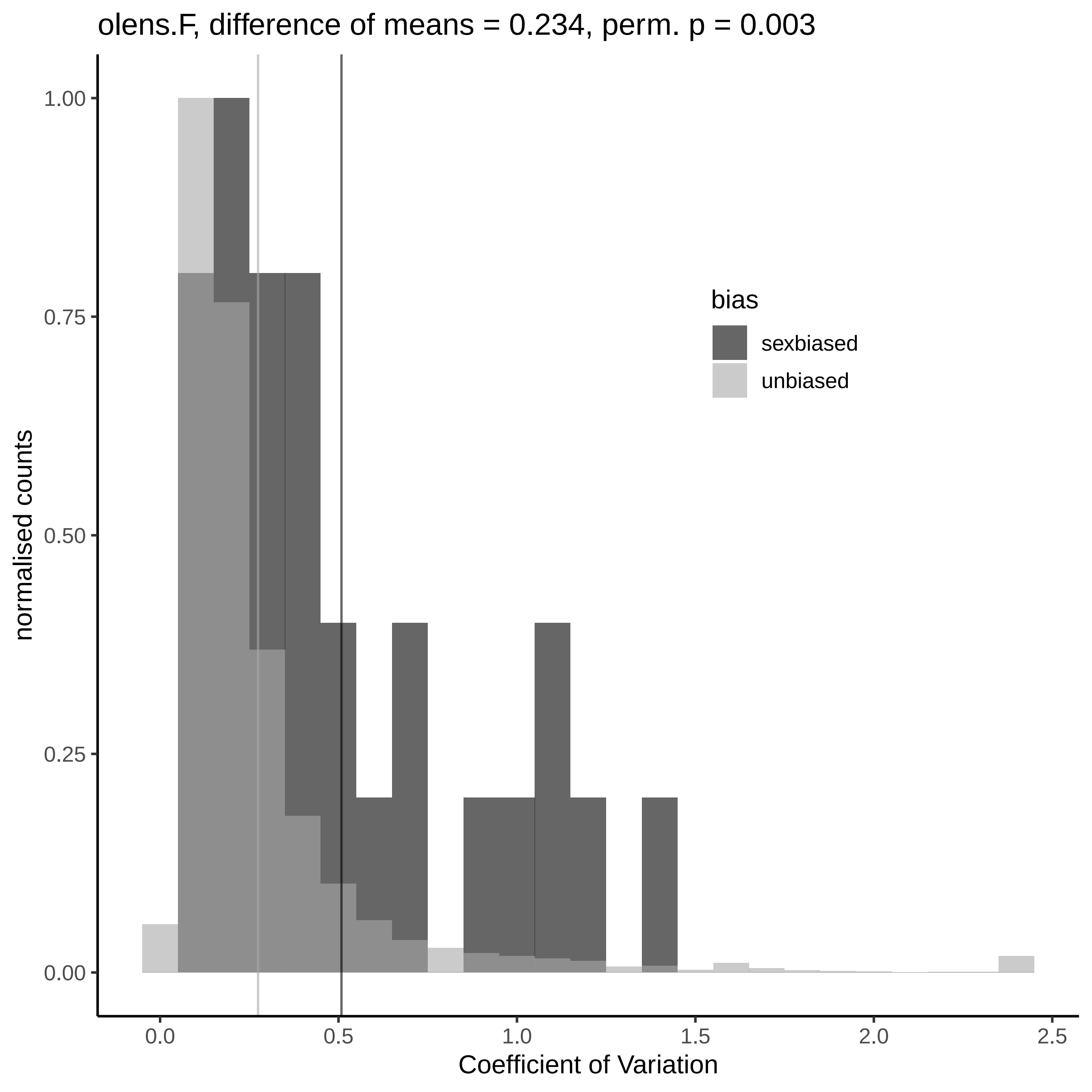
olens.F, difference of means = 0.234, perm. p = 0.003
normalised counts
1.00
0.75
0.50
0.25
0.00
0.0
0.5
1.0
1.5
2.0
2.5
Coefficient of Variation
bias
sexbiased
unbiased

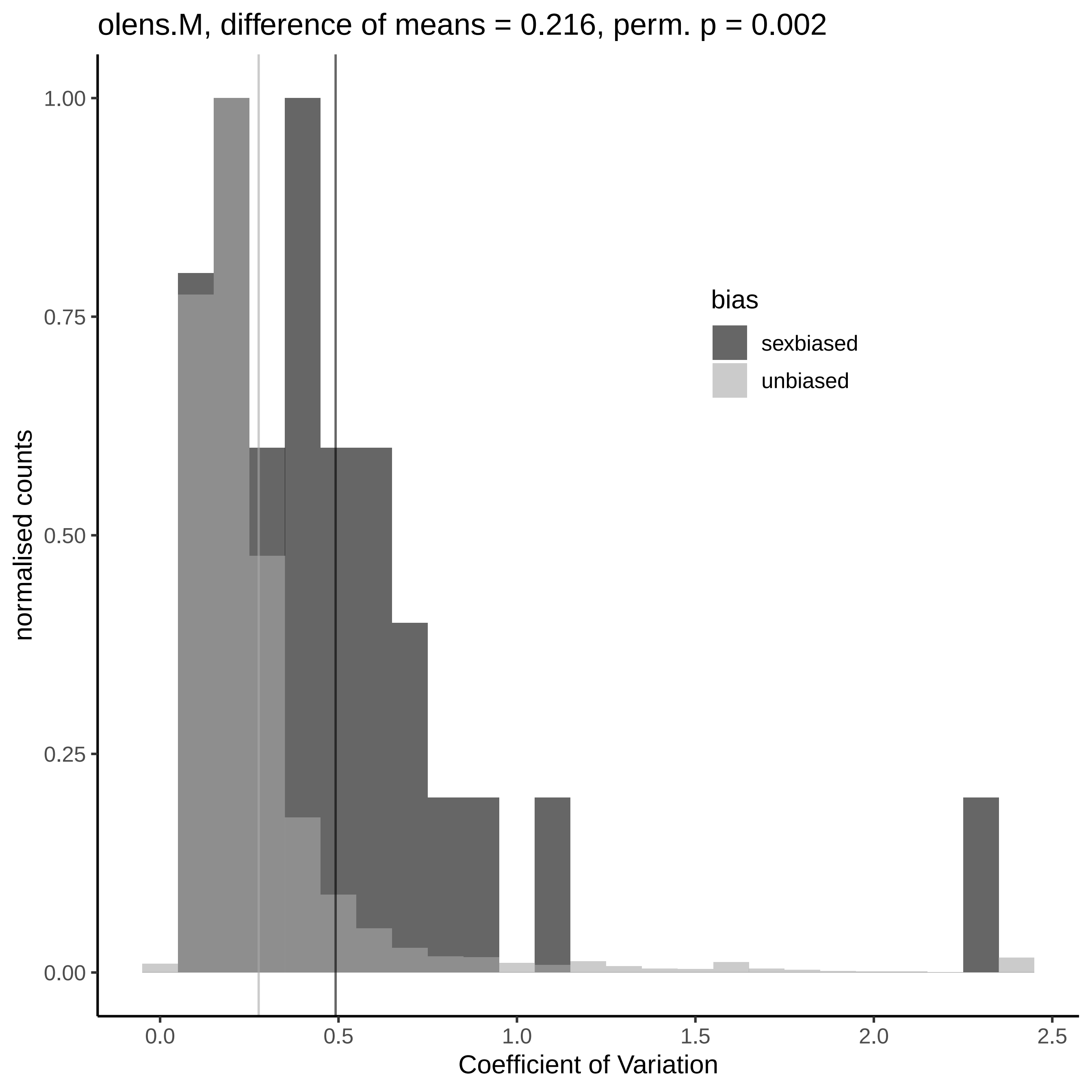
olens.M, difference of means = 0.216, perm. p = 0.002
normalised counts
1.00
0.75
0.50
0.25
0.00
0.0
0.5
1.0
1.5
2.0
2.5
Coefficient of Variation
bias
sexbiased
unbiased

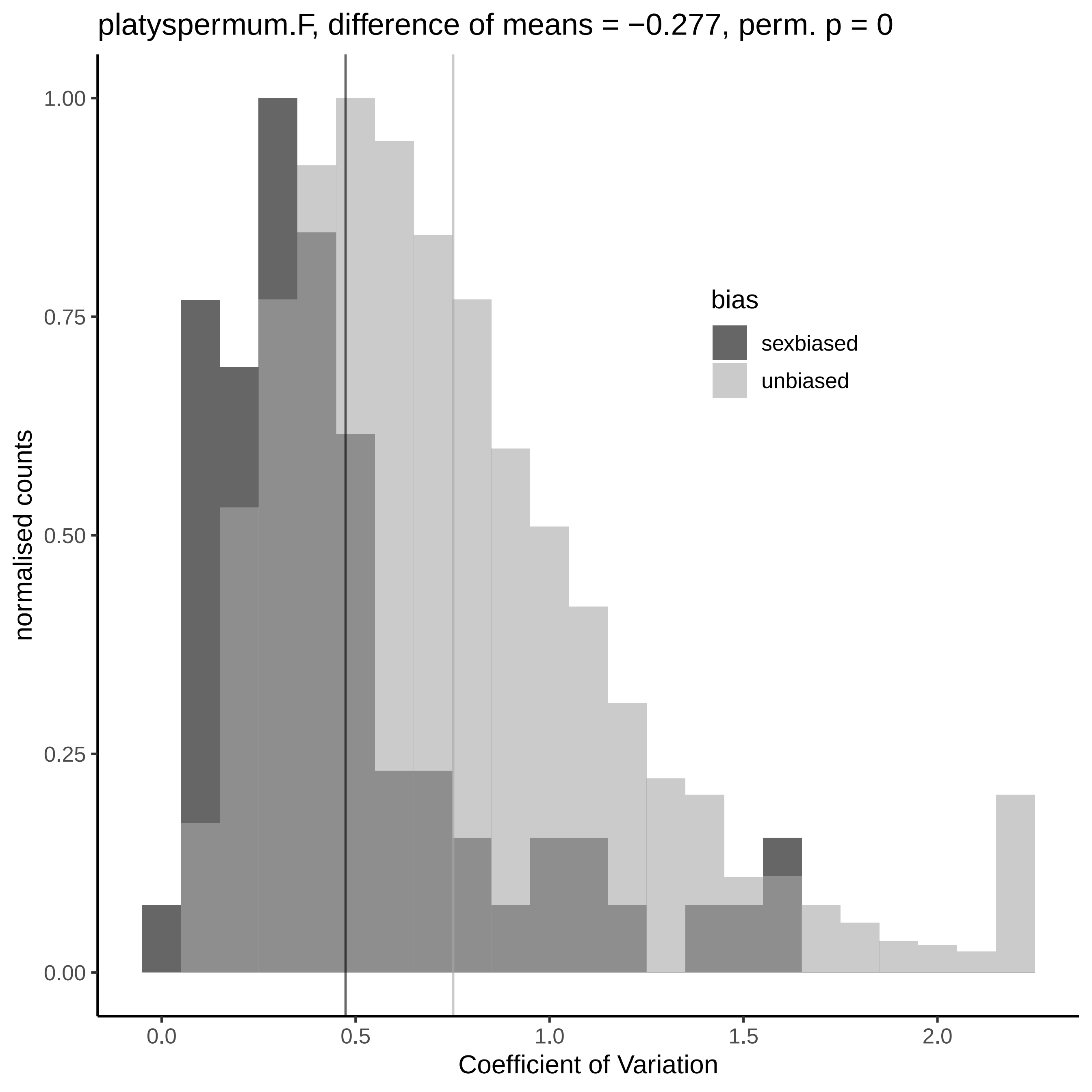
platyspermum.F, difference of means = −0.277, perm. p = 0
normalised counts
0.00
0.25
0.50
0.75
1.00
0.0
0.5
1.0
1.5
2.0
Coefficient of Variation
bias
sexbiased
unbiased

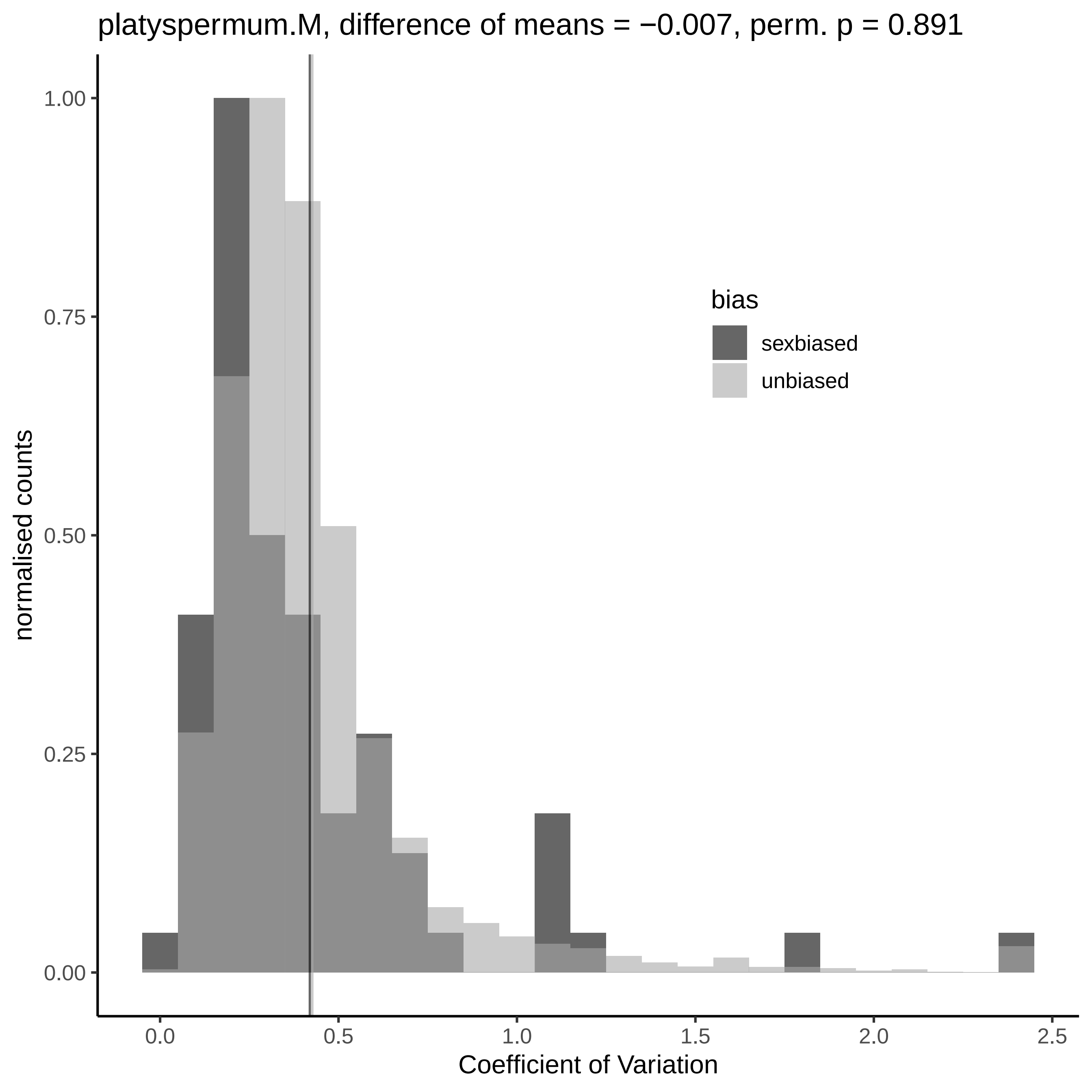
platyspermum.M, difference of means = −0.007, perm. p = 0.891
normalised counts
1.00
0.75
0.50
0.25
0.00
0.0
0.5
1.0
1.5
2.0
2.5
Coefficient of Variation
bias
sexbiased
unbiased

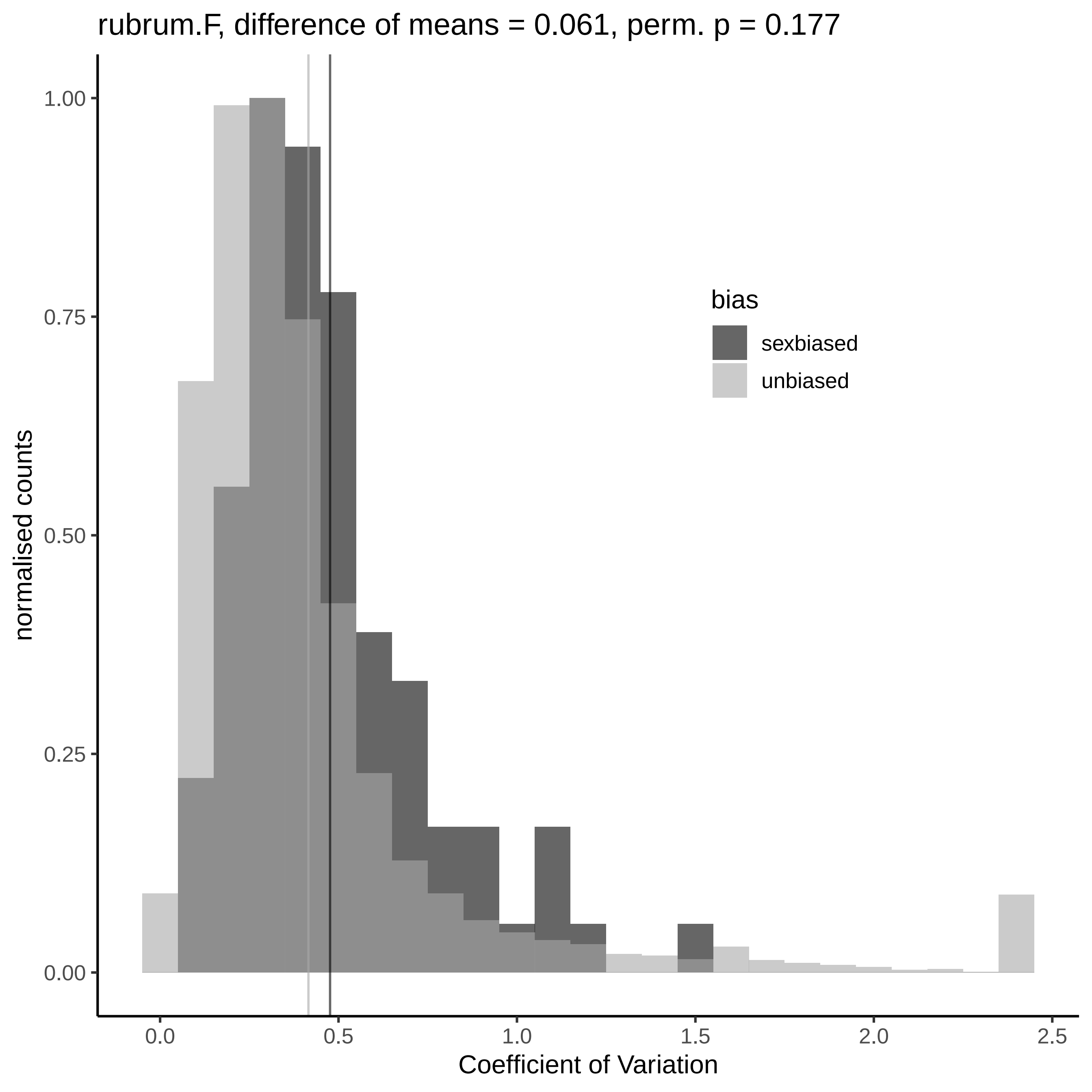
rubrum.F, difference of means = 0.061, perm. p = 0.177
bias
sexbiased
unbiased
normalised counts
0.00
0.25
0.50
0.75
1.00
0.0
0.5
1.0
1.5
2.0
2.5
Coefficient of Variation

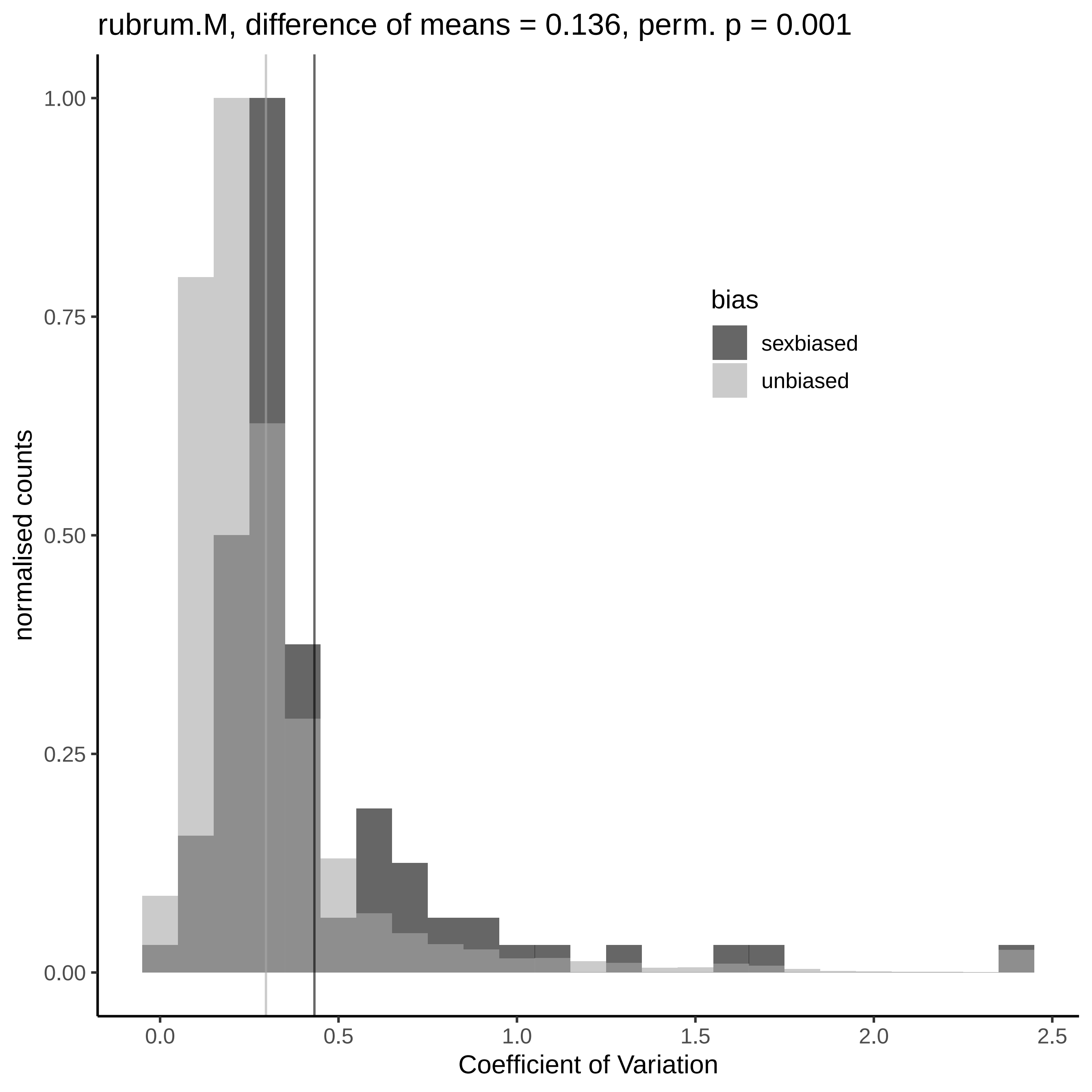

rubrum.M, difference of means = 0.136, perm. p = 0.001
normalised counts
1.00
0.75
0.50
0.25
0.00
0.0
0.5
1.0
1.5
2.0
2.5
Coefficient of Variation
bias
sexbiased
unbiased

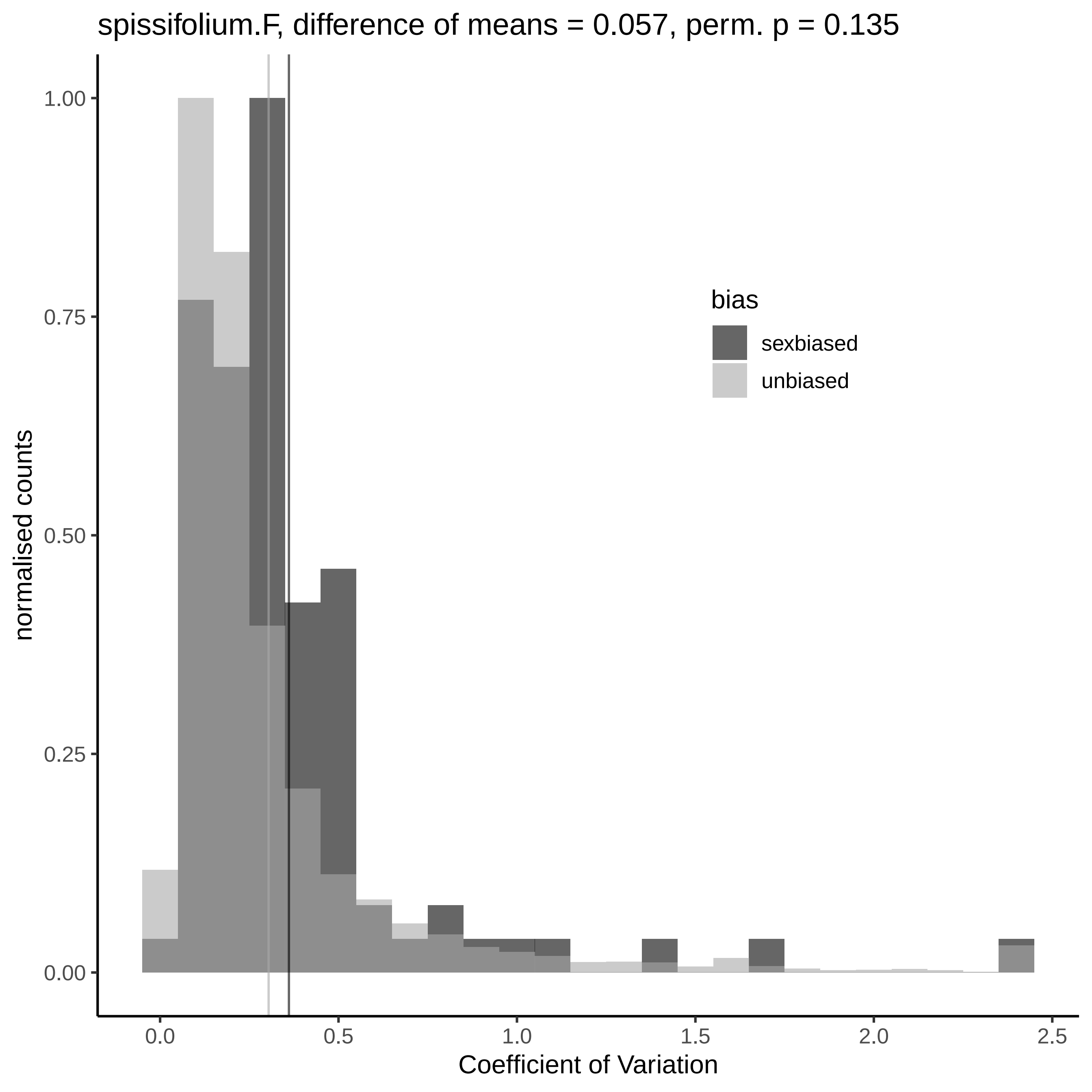

spissifolium.F, difference of means = 0.057, perm. p = 0.135
normalised counts
1.00
0.75
0.50
0.25
0.00
0.0
0.5
1.0
1.5
2.0
2.5
Coefficient of Variation
bias
sexbiased
unbiased

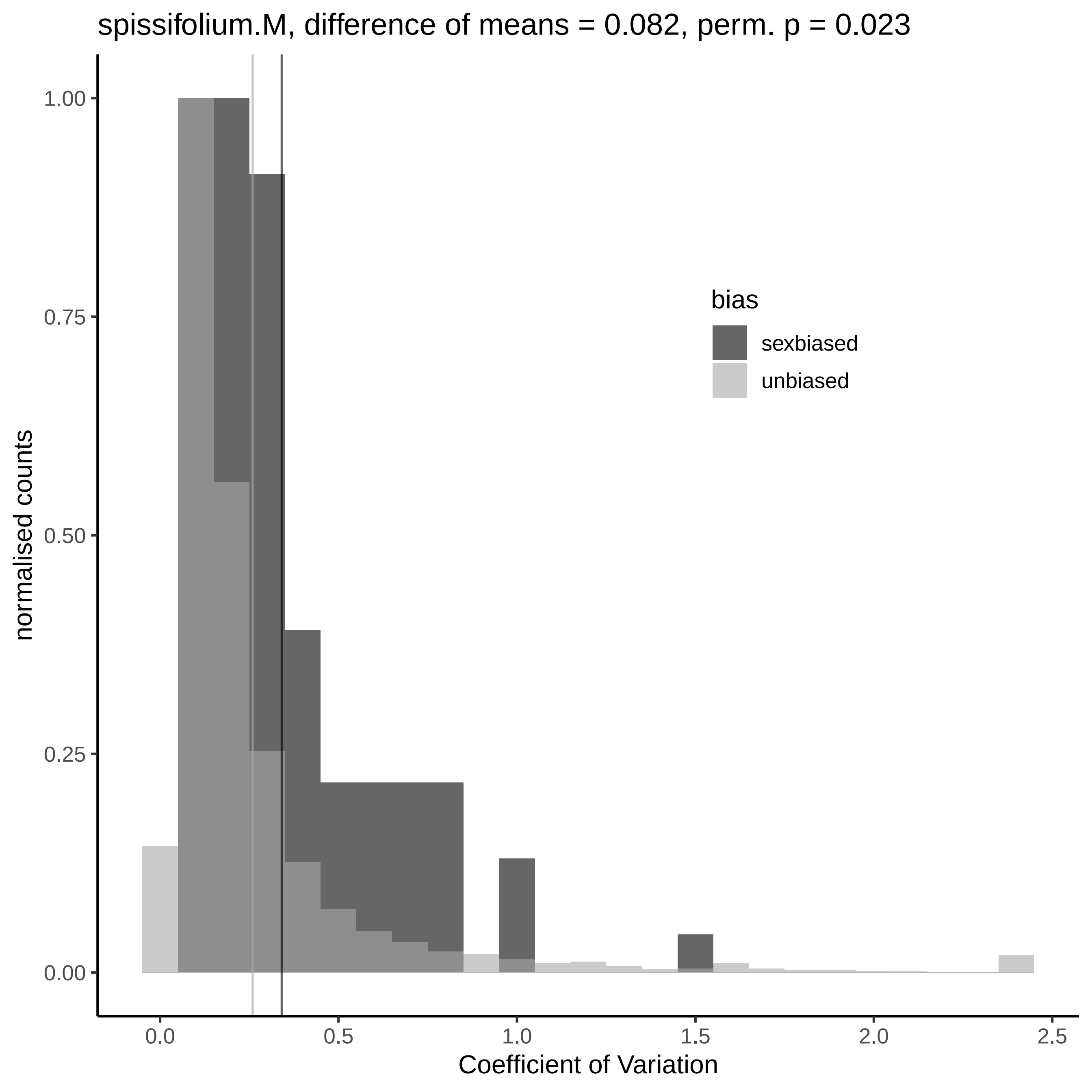
spissifolium.M, difference of means = 0.082, perm. p = 0.023
normalised counts
1.00
0.75
0.50
0.25
0.00
0.0
0.5
1.0
1.5
2.0
2.5
Coefficient of Variation
bias
sexbiased
unbiased

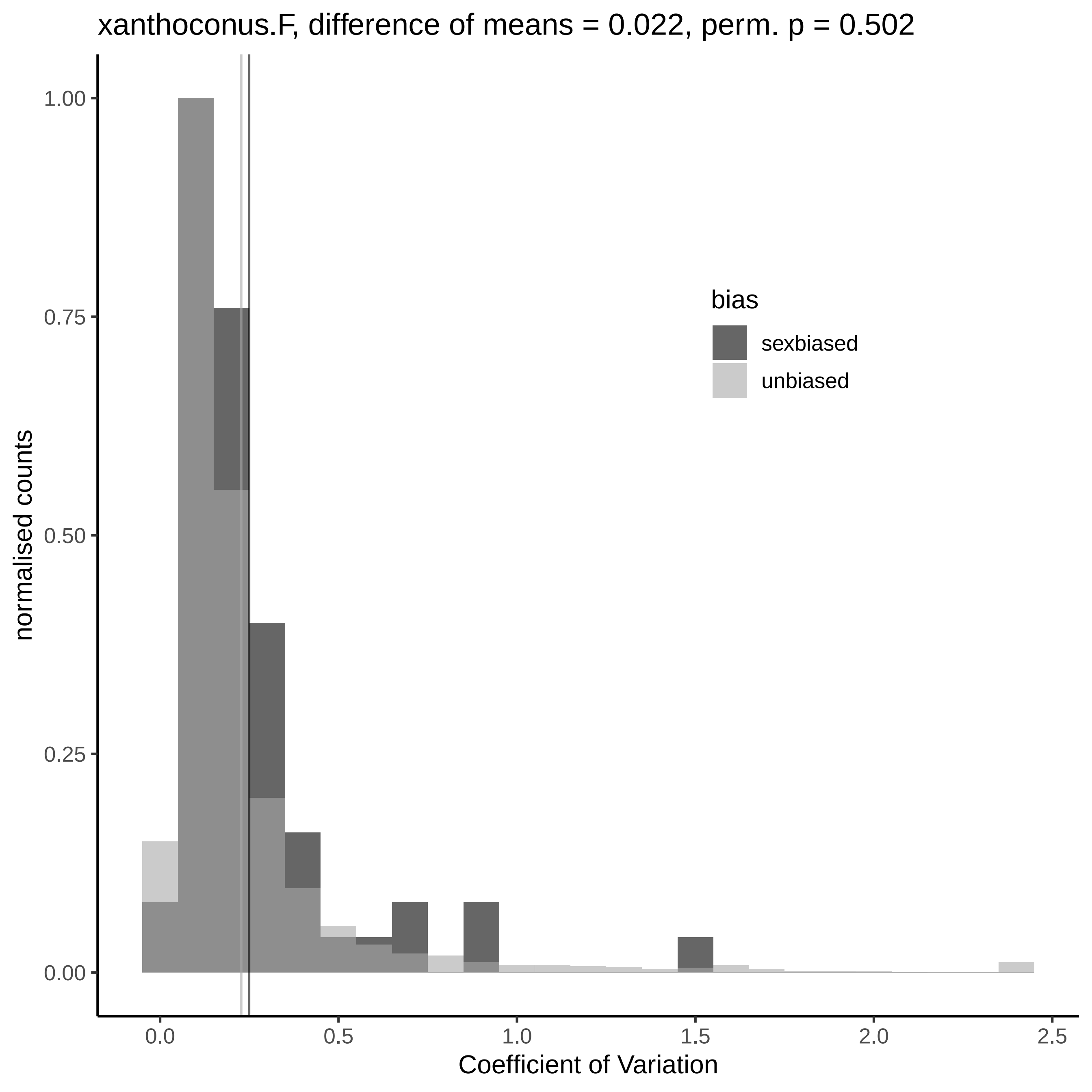
xanthoconus.F, difference of means = 0.022, perm. p = 0.502
normalised counts
1.00
0.75
0.50
0.25
0.00
0.0
0.5
1.0
1.5
2.0
2.5
Coefficient of Variation
bias
sexbiased
unbiased

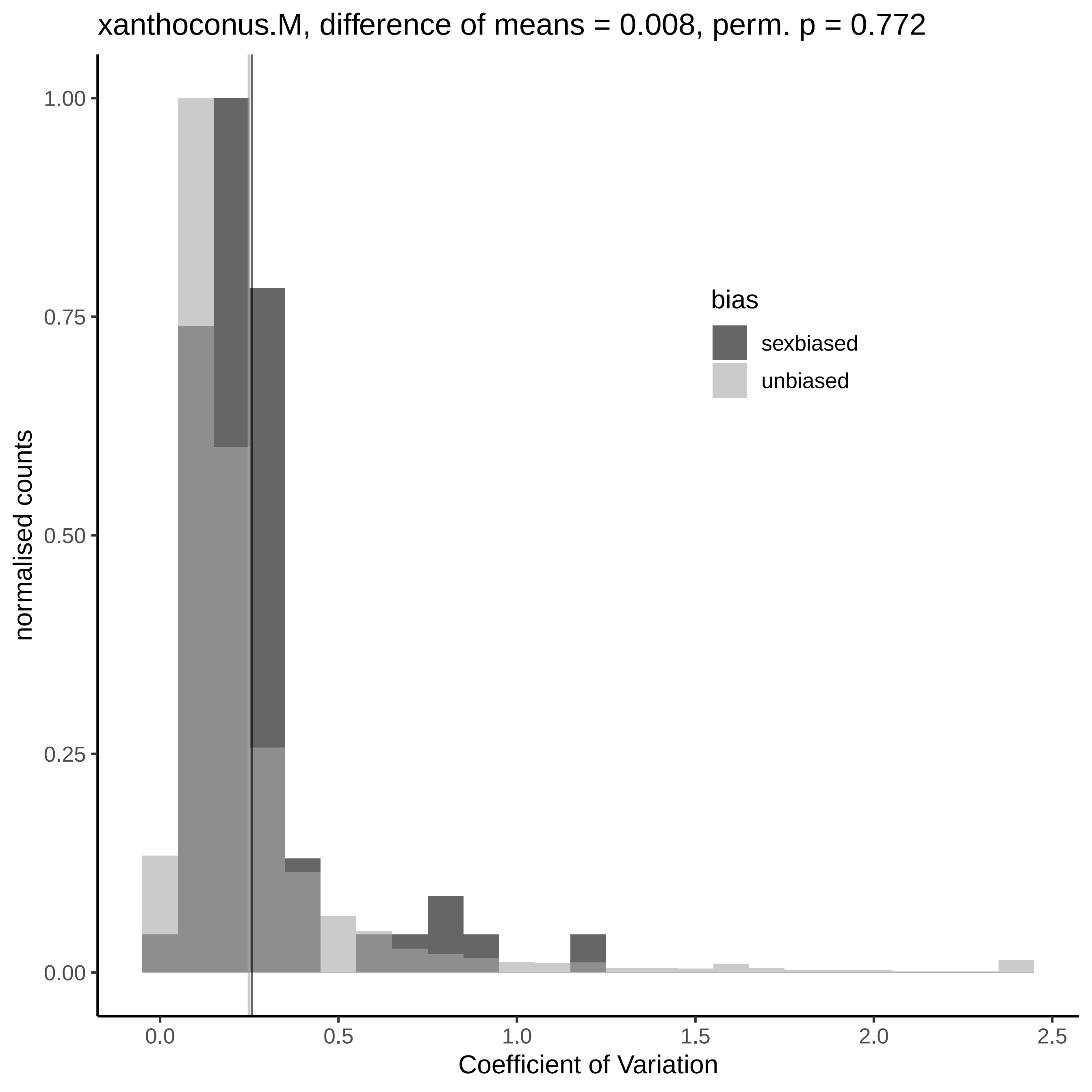
xanthoconus.M, difference of means = 0.008, perm. p = 0.772
normalised counts
1.00
0.75
0.50
0.25
0.00
0.0
0.5
1.0
1.5
2.0
2.5
Coefficient of Variation
bias
sexbiased
unbiased
