# Supplementary figures and images for: High rates of evolution preceded shifts to sex-biased gene expression in Leucadendron, the most sexually dimorphic angiosperms
Source: eLife. 2021 Nov 2;10:e67485. doi: 10.7554/eLife.67485 (PMC8635981; doi:10.7554/eLife.67485)

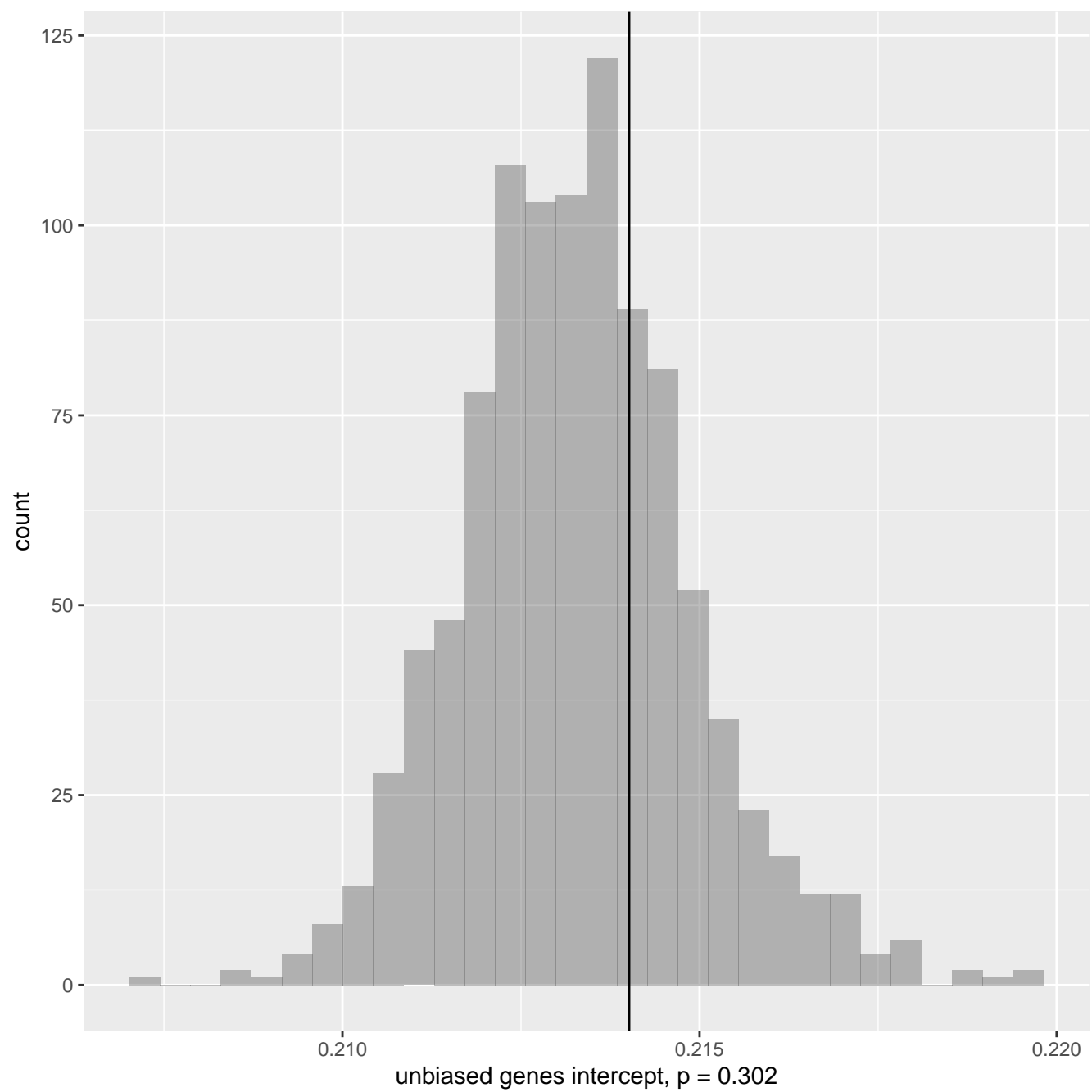

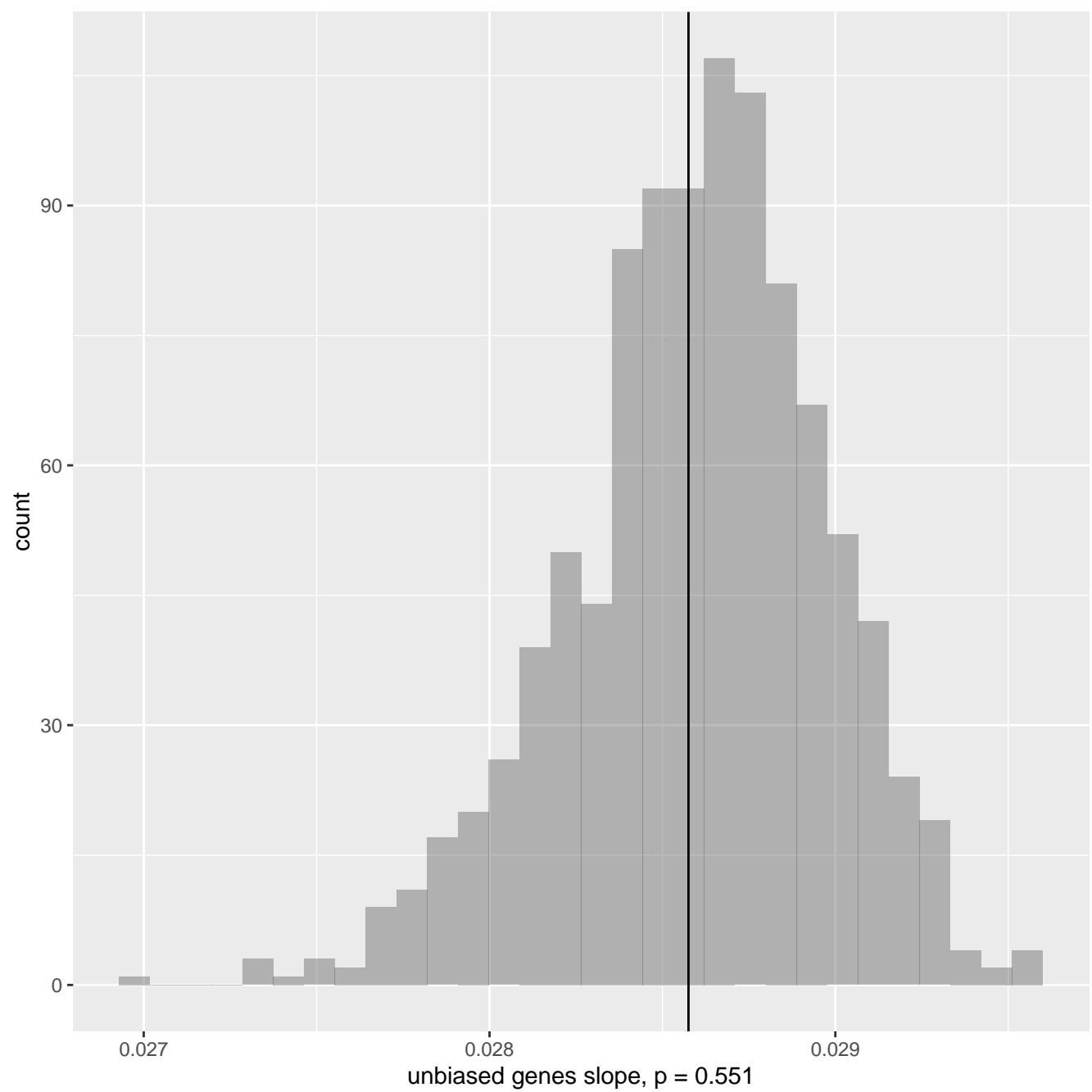

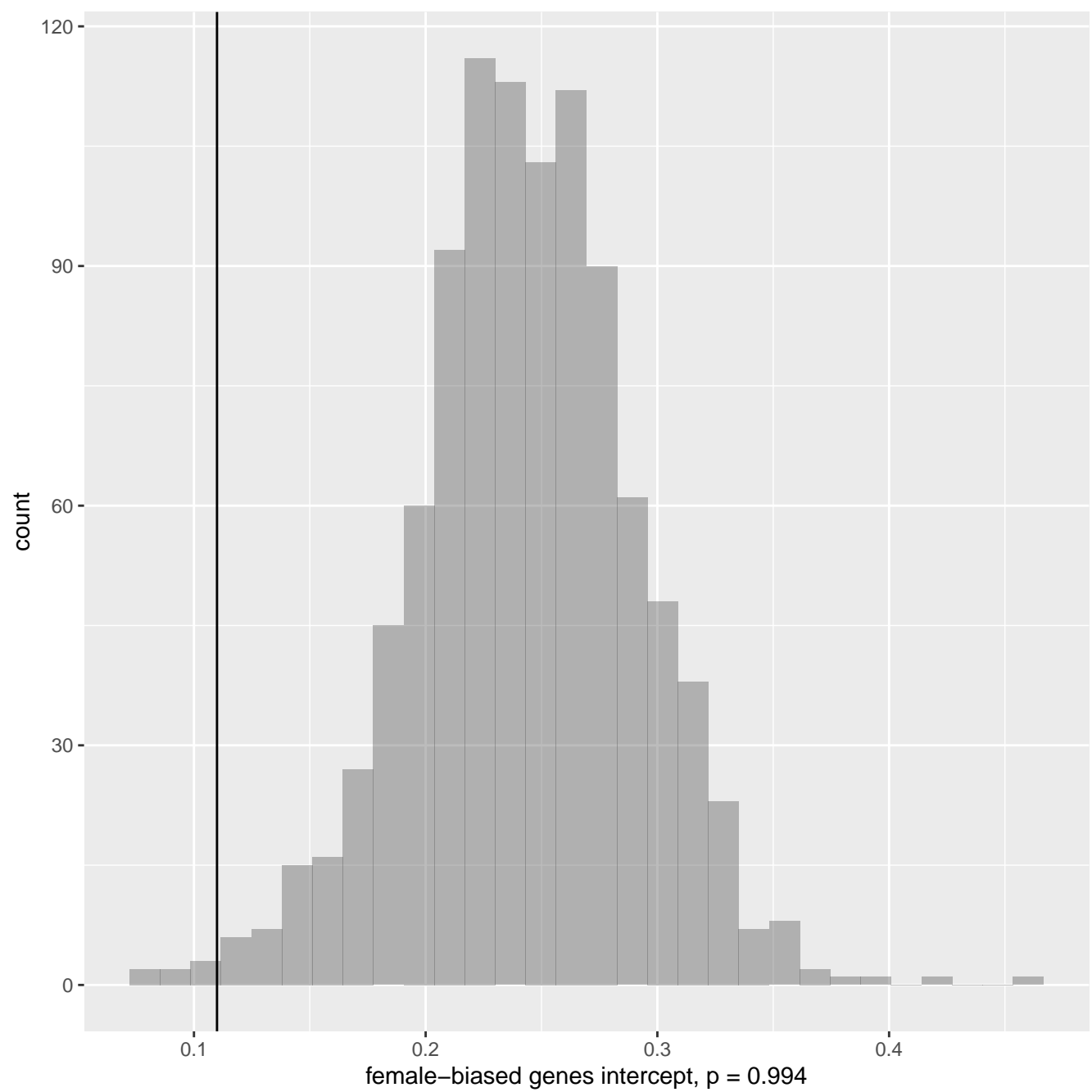

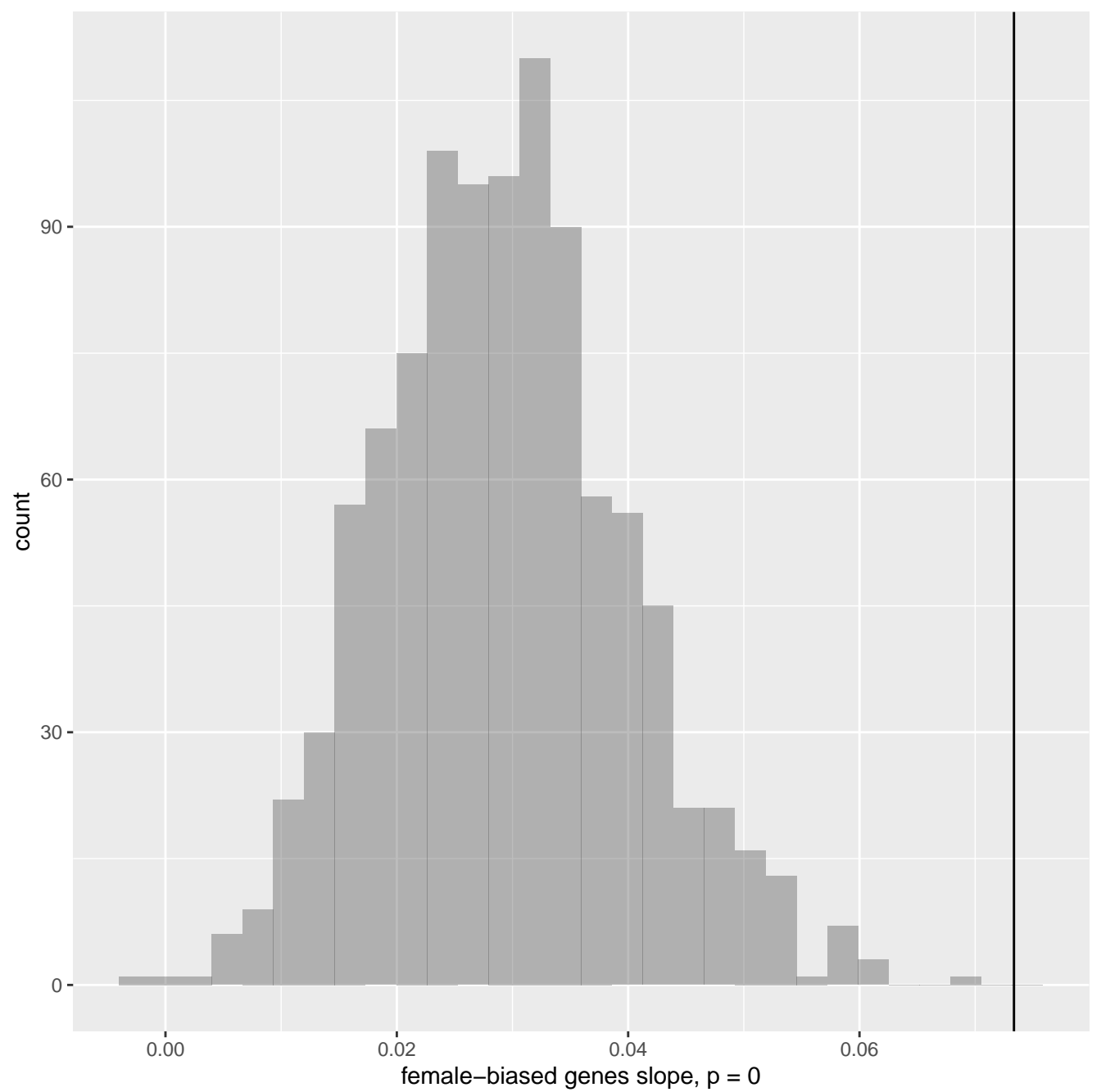

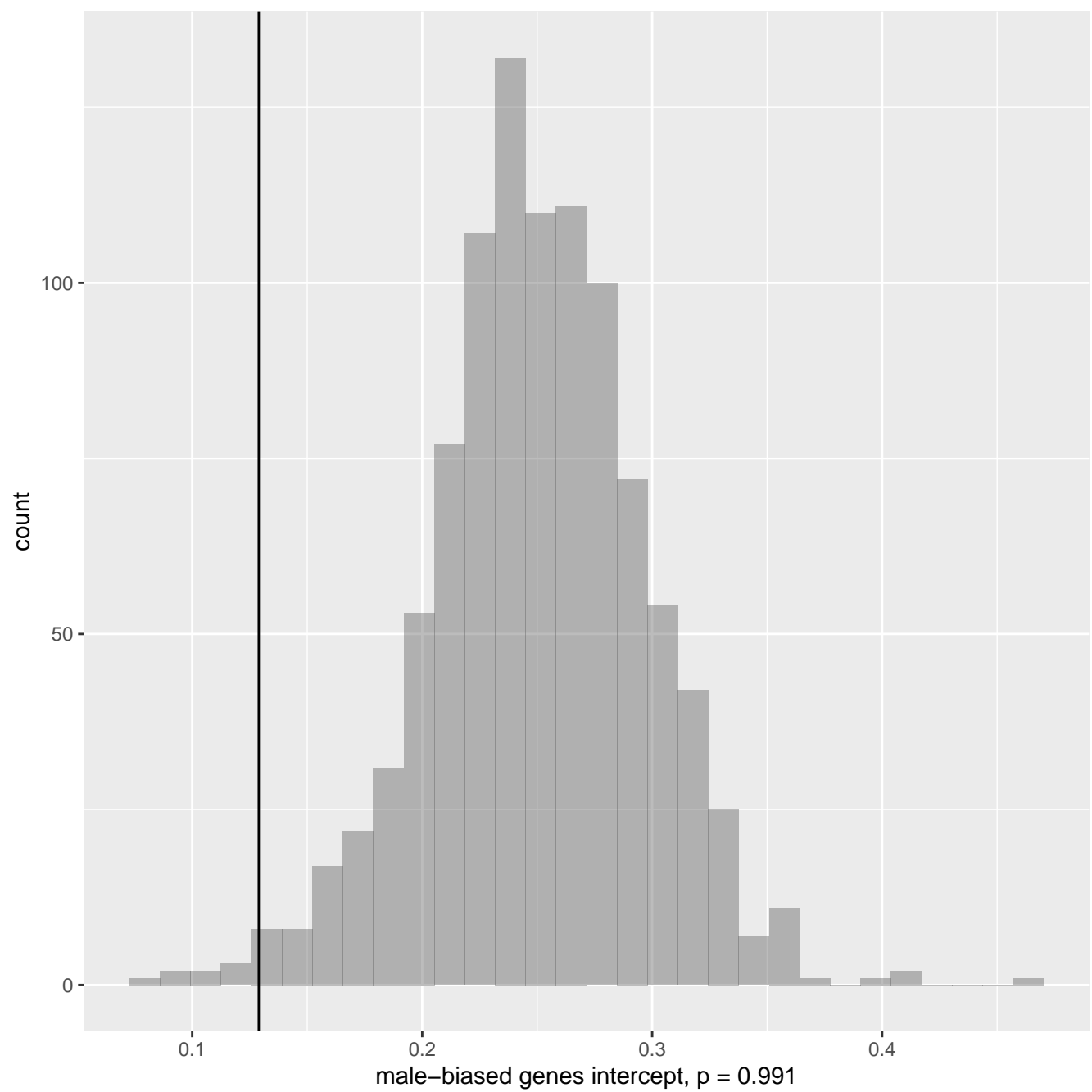

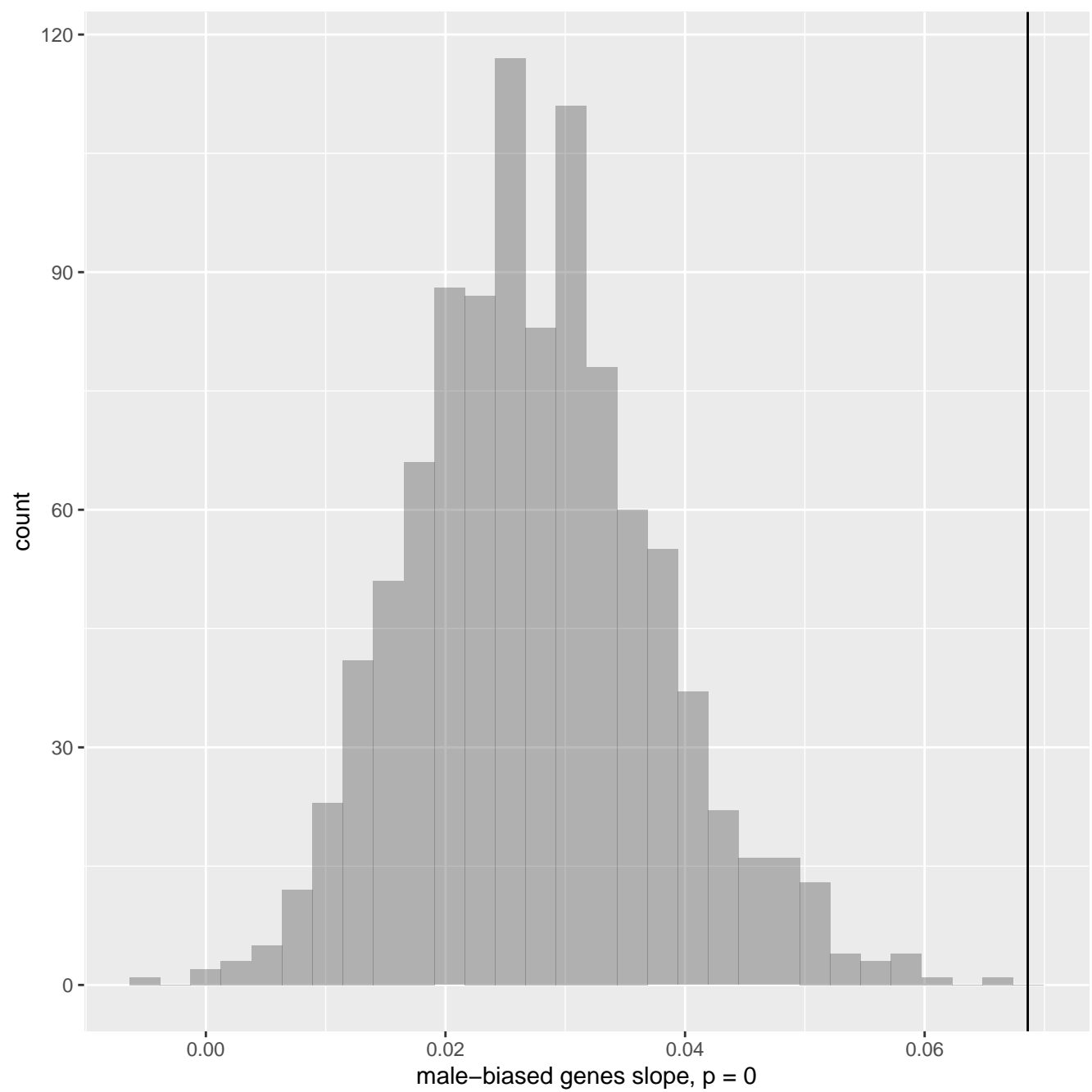

Supplement: Supplementary file 3. — Shown are the observed intercept and slope estimates, together with histograms of the same estimates for 1000 datasets in which the actual sex-biased genes were replaced by an equal number of unbiased genes that are matched to the expression noise level of sex-biased genes (i.e., within 95%–105% of their coefficient of variation in expression over all species and sexes). [file elife-67485-supp3.pdf]
